# Hierarchical hollow Fe_2_O_3_@MIL-101(Fe)/C derived from metal-organic frameworks for superior sodium storage

**DOI:** 10.1038/srep25556

**Published:** 2016-05-06

**Authors:** Chengping Li, Qian Hu, Yan Li, Hang Zhou, Zhaolin Lv, Xiangjun Yang, Lixiang Liu, Hong Guo

**Affiliations:** 1 School of Chemistry Science and Engineering, Yunnan University, Kunming 650091, Yunnan, China; 2 Institute for Integrative Nanosciences, IFW Dresden, Helmholtzstr. 20, 01069 Dresden, Germany

## Abstract

A facile generic template-free strategy is employed to prepare hierarchical hollow hybrid Fe_2_O_3_@MIL-101(Fe)/C materials derived from metal-organic frameworks as anode materials for Na-ion batteries. The intrinsic hollow nanostructure can shorten the lengths for both electronic and ionic transport, enlarge the surface areas of electrodes, and improve accommodation of the volume change during Na^+^ insertion/extraction cycling. Therefore, The stable reversible capacity of Fe_2_O_3_@MIL-101(Fe)/C electrode is 710 mAhg^−1^, and can be retained at 662 mAhg^−1^ after 200 cycles with the retention of 93.2%. Especially, its overall rate performance data confirm again the importance of the hierarchical hollow structures and multi-elements characteristics toward high capacities in both low and high current rates. This general strategy may shed light on a new avenue for fast synthesis of hierarchic hollow functional materials for energy storage, catalyst, sensor and other new applications.

The sodium-ion batteries (SIBs) recently have attracted great interest for large-scale energy storage in renewable energy and smart grid applications due to the cheap raw materials and its decent energy densities^1^,^2^,^3^,^4^. However, compared to Li-ion batteries (LIBs), Na ions (0.102 nm in radius) are about 34% larger than that of Li ions (0.076 nm in radius), which makes it difficult to find a suitable host material to allow reversible and rapid ion insertion and extraction^5^,^6^,^7^. And thus, the current SIBs face severe challenges from low energy density and poor cycling stability since sodium intercalation induces anisotropic volume expansion, which triggers large voltage polarization and insufficient contact with the current collector. Therefore, development of good performance electrode materials having high capacities and long term stability is essential to successful commercialization of batteries. The current anode material studies focus on carbonaceous materials^8^,^9^,^10^. However, their reversibility and rate capacity are still limited. In comparison, transition metal oxides based on conversion reactions, such as Fe, Co, Ni, Cu, Mn and Mo, are proposed for the anode materials of SIBs, and these oxides can react with Na^+^ through a one-step conversion reaction^11^,^12^,^13^,^14^:


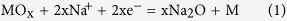


Of which, iron oxides attractive anodes due to their high theoretical capacity and low cost. Komaba *et al.* first testified the nanocrystallized α -Fe_2_O_3_ and Fe_3_O_4_ in the sodium salt electrolyte, however, the obtained sodium storage capacity was rather low^15^. Most recently, Fe_2_O_3_@GNS exhibited an initial capacity of 535 mAhg^−1^ and 75% capacity retention after 200 cycles at 100 mAg^−1^ A full Na ion cell based on the Fe_3_O_4_/C anode and Na[Ni_0.25_Fe_0.5_Mn_0.25_]O_2_ layered cathode delivered a capacity of 130 mAhg^−1^ and 76.1% capacity retention after 150 cycles^16^. However, the reversible capacities of most of above metal oxides are not higher than 600 mAhg^−1^ and the initial coulombic efficiency is low.

To solve this problem, allowing the electrochemical reaction to proceed in a hybrid matrix of distinct material systems, such as metal oxides integrated with carbon, is an effective way to control the volume changes. The coupling of two species could render the iron oxides with rich redox reactions and improve electronic conductivity. As a result, the geometric configuration and cycling stability of electrode are improved. For example, the recent porous γ -Fe_2_O_3_@C nanocomposites anode has been characterized by Chen’s group exhibited high sodium storage capacity and outstanding cyclability^17^. Another popular way to improve the electrochemical performance has been focused on controlling the volumetric change by using small particles with various morphologies including nanotube, nanowall, nanosheet, nanobowl, nanocone, and porous solid, of which hollow particles are of particular interest for reversible ion storage because of their short ion diffusion and good toleration for volume change during cycling. This design was former widely used in Li^+^ batteries^18^,^19^,^20^. In fact, hollow micro/nanoscale metal oxides are of great interest due to well-defined interior voids, high specific surface area, low density, accommodate volume change without pulverizing compared with that of solid counterparts of the same size, and thus make them attractive materials for applications such as dye-sensitized solar cells, catalysis, chemical sensors, drug delivery, and energy conversion and storage systems. For example, our previous prepared hollow cage-bell Ag@TiO_2_ exhibit excellent lithium-ion storage, photocatalytic and catalytic performance^21^. X. W. Lou and co-workers synthesized hollow microcubes of metal sulfides for enhanced lithium storage^22^. Therefore, a general approach to rationally fabricate hollow hybrid structural materials is still lacking and it is desirable to obtain the materials through more facile, economic and environment-friendly process. Metal–organic frameworks (MOFs), a new class of organic-inorganic hybrid functional materials with high porosity, large surface area and morphology can be easily tuned upon selection of different metal ions and organic bridging ligands^23^. Recently, MOFs are proved to be an effective template for preparing hollow transition metal oxides by thermal decomposition, because the porosity and long-range ordering of MOFs can offer a fast and convenient access for incoming and leaving small molecules and ions in the transformation process. Though these procedures are effective, each design strategy alone always leads to limited improvement in the electrochemical properties. And thus, the developing of a facile, scalable and controllable fabrication of hierarchic hybrid iron oxides materials with satisfactory cycling ability and high capacity is still highly desirable for SIBs. Besides, to our best knowledge, reports on the fast synthesis of hollow structures derived from MOFs used for Na^+^ batteries are quite rare, and it can be an advantage for chemists to elaborate possible new constructions from all chemical components without any time-restricted conditions.

Herein, we chose Fe-O-C hybrid nanostructure to demonstrate our concept and propose a facile fast strategy to prepare hybrid structures from MOFs. In this work, the advantage of novel hollow structures from MOFs and the virtue of hybrid matrix of distinct material systems are well integrated to solve the problem. Compared with conventional methods produced metal oxides nano electrodes, the hollow nanostructures prepared via MOFs have relatively high surface area and a stable hollow configuration without the destructive effect of template removal on product morphology. The hollow structures can render much contact area between active components and Na ions in the process of electrochemical reaction. Meanwhile, it may help the electrode to accommodate large volume change without pulverizing. Futhermore, the unique structure can shorten ionic/electronic diffusion length and provide efficient channels for mass transport^24^,^25^,^26^. The multi-components allow the electrochemical reaction to proceed in a hybrid matrix of distinct material systems. Moreover, the coupling of carbon could render the Fe_2_O_3_ with rich redox reactions and improve electronic conductivity. As a result, the geometric configuration and outstanding electrochemical performance of hierarchical hollow γ -Fe_2_O_3_@C structures from MOFs are anticipated to manifest outstanding electrochemical performance.

## Results and Discussion

### Structure and morphology of hollow Fe_2_O_3_@MIL-101(Fe)/C

**Figure 1:**
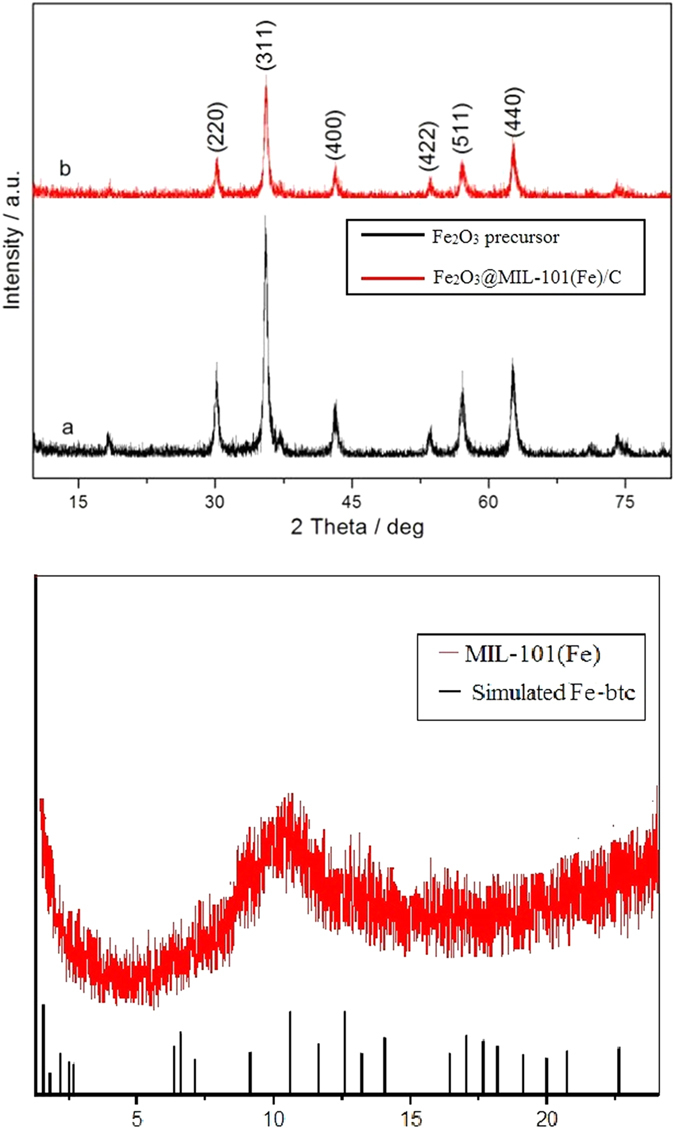
(**a**) X-ray diffraction (XRD) patterns of Fe_2_O_3_ precursor and hollow Fe_2_O_3_@MIL-101(Fe)/C sample corresponding to curve a and b, respectively. (**b**) XRD patterns of Fe-MOF in the 2θ range of 1–23°.

The crystallographic structure and phase purity of Fe_2_O_3_ precursor and hollow Fe_2_O_3_@MIL-101(Fe)/C magnetic sample were analyzed by XRD, as shown in Fig. 1a. All the diffraction peaks can be indexed to the γ -Fe_2_O_3_ (JCPDS card no. 39–1346). No other impurity peaks are observed, indicating a complete thermal conversion of the MOFs precursors into Fe_2_O_3_ nanostructures. Detailed analysis of the peak broadening of the (3 1 1) reflection of Fe_2_O_3_@MIL-101(Fe)/C using the Scherrer equation indicates an average crystallite size ca. 5 nm, suggesting that these particles are composed of nanocrystal subunits. Figure 1b presents X-ray diffraction (XRD) patterns of the Fe-MOFs and their simulated results which reveal that the composition of the precursors is MIL-101(Fe). All the diffraction peaks of the formed Fe-btc MOFs in the 2θ range of 1–23° are in agreement with the results.

**Figure 2:**
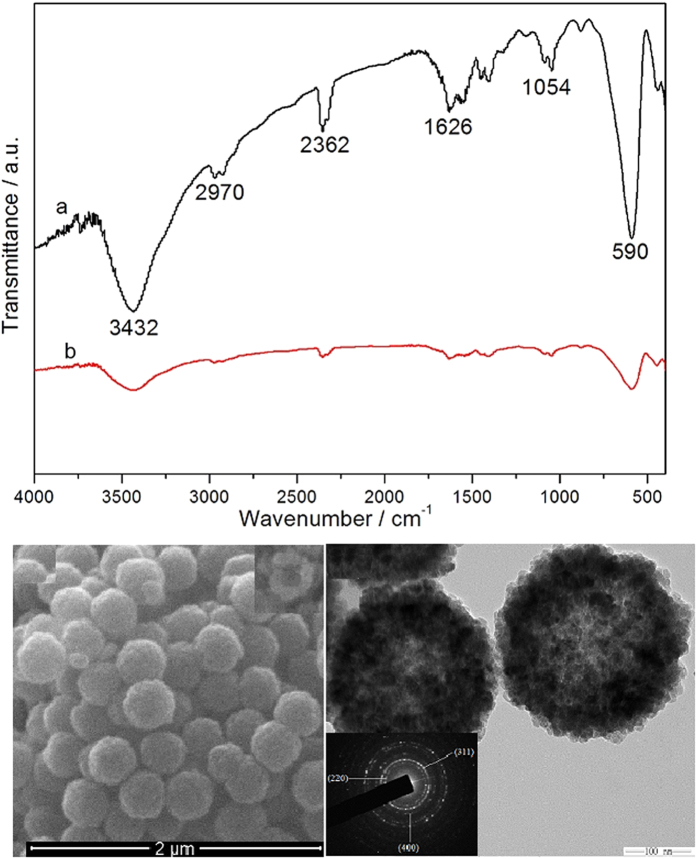
(**a**) FTIR spectra of prepared hollow Fe_2_O_3_ precursor and Fe_2_O_3_@MIL-101(Fe)/C. SEM (**b**) and TEM (**c**) images of hollow Fe_2_O_3_ precursor. The inset in (**c**) is the selected area electron diffraction (SAED).

**Figure 3:**
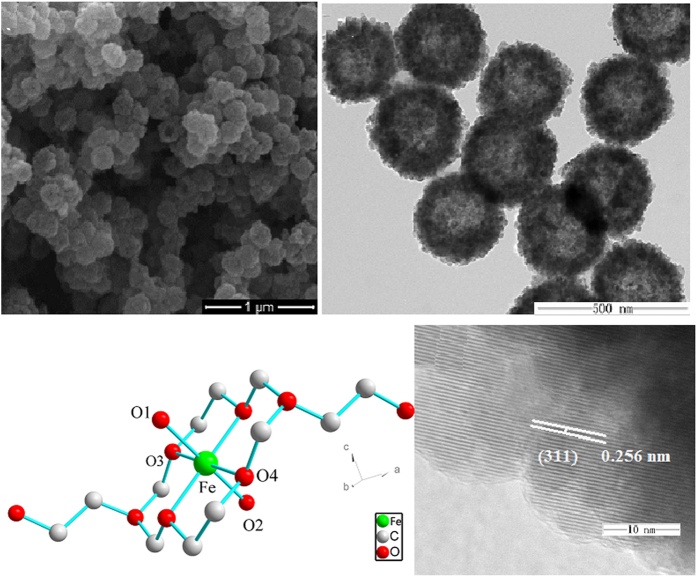
SEM (**a**), TEM (**b**), the residual atomic space structure (**c**), and HRTEM (**d**) of the micrographs of as-prepared hollow porous Fe_2_O_3_@MIL-101(Fe)/C.

The FTIR spectrum images of the prepared hollow structured Fe_2_O_3_@MIL-101(Fe)/C magnetic sample and its Fe_2_O_3_ precursor are shown in Fig. 2a. The broad absorption peaks from 3432 to 2362 cm^−1^ are associated with the stretching vibrations of the -OH group of absorbed water molecules and absorption of CO_2_ in the air, and the peak of 1626 cm^−1^ is assigned to the bending vibrations of the water molecules. For the Fe_2_O_3_@MIL-101(Fe)/C, the spikes from 1617 to 1000 cm^−1^ are assigned to the asymmetric and symmetric stretching vibrations of the carbonyl group of organics. The strongest broad peaks in the range of 400–1000 cm^−1^ are contribution from the face-centered cubic phase Fe-O. The peak intensity of Fe-O bond for final products is different from that of precursor, implying the structure of prepared sample has some discrepancy with its precursor. The SEM and TEM images of the prepared hollow Fe_2_O_3_ precursors are shown in Fig. 2b,c. It is obvious that the precursors are hollow sub-microsphere uniformly with average diameter of ca. 300 nm according to Fig. 2b. TEM (Fig. 2c) reveals that these sub-microspheres exhibit hollow structures obviously. Its selected-area electron diffraction (SAED) pattern (Fig. 2c) reveals the diffraction rings 1–3 are indexed to (2 0 0), (3 1 1), and (4 0 0) diffraction of Fe_2_O_3_, respectively. This result is in good agreement with XRD analysis. The unique hollow porous morphologies of Fe_2_O_3_@MIL-101(Fe)/C are characterized by SEM, TEM and HR-TEM, and the results are illustrated in Fig. 3a–d. It is clear that the prepared Fe_2_O_3_@MIL-101(Fe) keeps the original morphology of its precursors (Fig. 3a). The TEM image in Fig. 3b shows the hollow structure, which is a visible hollow interior structure obviously. Especially, a typical structure with well-defined interior and shell can also be detected and the thickness of shell of samples is ca. 150–200 nm. Furthermore, the surface of samples exhibits porous frame, which hierarchical structure is resulted from MOFs. Figure 3c is the molecular structure of Fe-MOFs. The lattice fringe is observed obviously, and the lattice spacing (0.256 nm) agrees with Fe_3_O_4_ (3 1 1) plane spacing from Fig. 3d. The size of as-synthesized Fe_2_O_3_@MIL-101(Fe)/C are much smaller than those reported by Zhu very most recently^27^.

**Figure 4:**
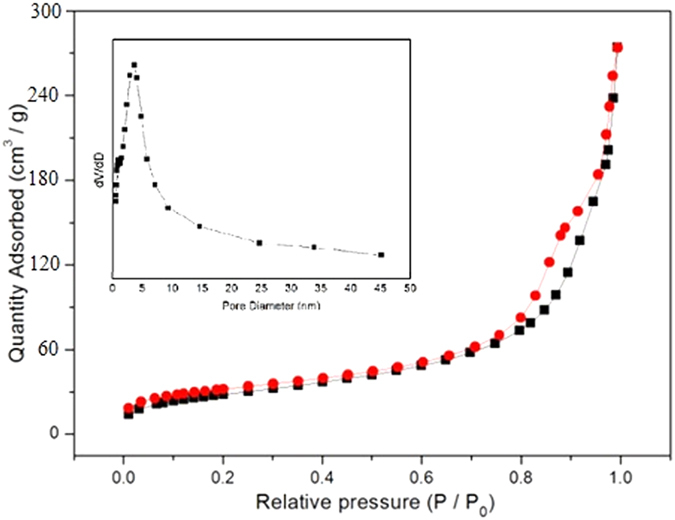
N_2_ adsorption/desorption isotherm (77 K) curve for hollow porous magnetic Fe_2_O_3_@MIL-101(Fe)/C.

The N_2_ adsorption/desorption isotherms and the pore size distribution of the obtained hollow porous hollow Fe_2_O_3_@MIL-101(Fe)/C are shown in Fig. 4. The isotherms are identified as type IV, which are the characteristic isotherm of mesoporous materials. The pore size distribution data indicates that average pore diameters of the product are in the range of 3–8 nm. The BET surface area of the sample is 123.15 m^2^ g^−1^. Remarkably, the specific surface area is far higher than most of the previously reported transition metal oxides microsphere products^28^,^29^,^30^. The single-point total volume of pores at P/P0 =  0.975 is 0.425 cm^3^g^−1^. These indicate that the prepared samples have a loose mesoporous structure. This structure can not only keep the nano effect of active components but also help to buffer the volume changes of hollow porous Fe_2_O_3_@MIL-101(Fe)/C electrodes during electrochemical reaction.

### Electrochemical characterizations of hierarchic hollow Fe_2_O_3_@MIL-101(Fe)/C

**Figure 5:**
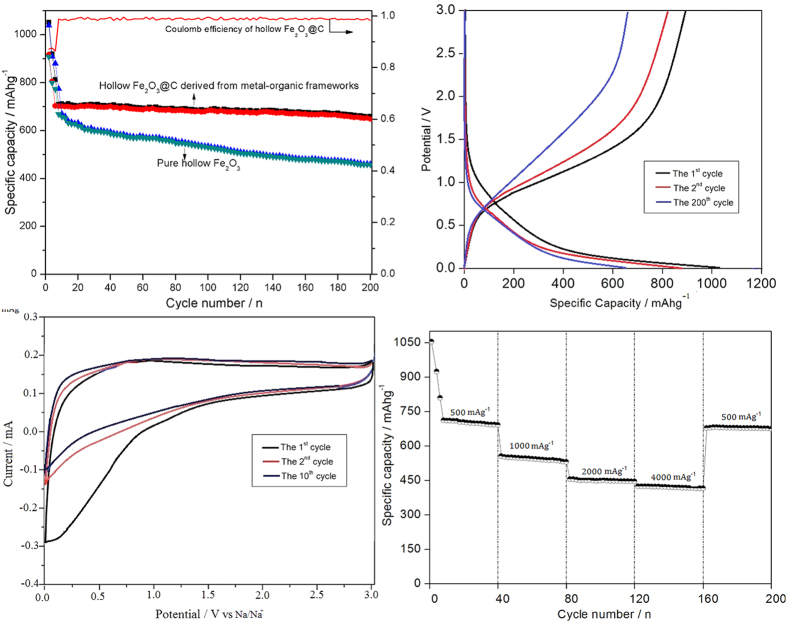
Electrochemical performance of prepared hierarchical hollow Fe_2_O_3_@MIL-101(Fe)/C electrodes: (**a**) The cycling performance of bare Fe_2_O_3_ and Fe_2_O_3_@MIL-101(Fe)/C measured at 200 mAg^−1^. (**b**) charge/discharge curves of Fe_2_O_3_@MIL-101(Fe)/C electrode for the 1^st^, 2^nd^, and 200^th^ cycle at current density of 200 mAg^−1^. (**c**) Cyclic voltammetry plots of Fe_2_O_3_@MIL-101(Fe)/C electrode at the scan rate of 0.05 mV s^−1^. (**d**) Rate capability of Fe_2_O_3_@MIL-101(Fe)/C electrode from 500 mAg^−1^ to 4000 mAg^−1^ for 200 cycles. Electrode potential range of 0.05–3.0 V vs. Na/Na^+^.

The electrochemical performance of the hollow Fe_2_O_3_ and Fe_2_O_3_@MIL-101(Fe)/C hybrid materials used for Na-ion anodic materials is investigated. The cycling performance profiles of Fe_2_O_3_ and Fe_2_O_3_@MIL-101(Fe)/C electrodes at constant current density of 200 mAg^−1^ are shown in Fig. 5a. The initial charge capacities are 1040 and 1052 mAhg^−1^ for bare Fe_2_O_3_ and Fe_2_O_3_@MIL-101(Fe)/C with corresponding coulombic efficiencies of 87.6% and 87.1%, respectively. The stable charge capacity of Fe_2_O_3_@MIL-101(Fe)/C electrode is 710 mAhg^−1^, and can be retained at 662 mAhg^−1^ after 200 cycles with the retention of 93.2%. The coulombic efficiencies are always over 99.6% except for the first 4 cycles at 200 mAhg^−1^. While the bare Fe_2_O_3_ samples present only 462 mAhg^−1^ after 200 cycles. Hence, Fe_2_O_3_@MIL-101(Fe)/C exhibits improved reversibility. The charge/discharge curves of hierarchic hollow Fe_2_O_3_@MIL-101(Fe)/C electrode for the first two and the 200^th^ cycle at constant current density of 200 mAg^−1^ are shown in Fig. 5b. It is noticeable that the Na-reaction potential in the Fe_2_O_3_ is quite lower than the Li-reaction potential. The electrolyte of Na^+^ batteries having lower decomposition potential compared with that of Li-ion batteries (LiPF_6_ system) should be responsible for the performance. In the initial discharge, the potential drops rapidly to a plateau of 1.1 V and then decreases gradually to the plateaus of approximately 0.4 V. The plateau is not so apparently, which is possible due to the presence of small nanoparticles of Fe_2_O_3_. The reaction can be described as equation (2) and the conversion of the iron oxide accompanied by the layer formation of solid electrolyte interface (SEI). which results are consistent with other research groups^1^,^7^,^12^.


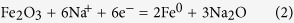


After 200 cycles, the capacity can also be kept at ca.660 mAhg^−1^, showing the excellent reversibility of electrode. The cyclic voltammograms (CVs) within the range of 0.05–3.0 V vs. Na/Na^+^ at the scan of 0.05 mVs^−1^ are shown in Fig. 5c. The first CV plot is different from those of the subsequent scans, which is attributed to the irreversible formation of a solid electrolyte interface (SEI). In the subsequent cycles, a broad cathodic peak at 0.67 V is attributed to the insertion-extraction into/from the crystal structure of Fe_2_O_3_^7^. These results are consistent with CV analysis above and other reports^7^,^12^. Compared with LIBs, the redox peaks is weak because of the large size, heavy mass, and poor mobility of Na^+^^31^,^32^.

**Figure 6:**
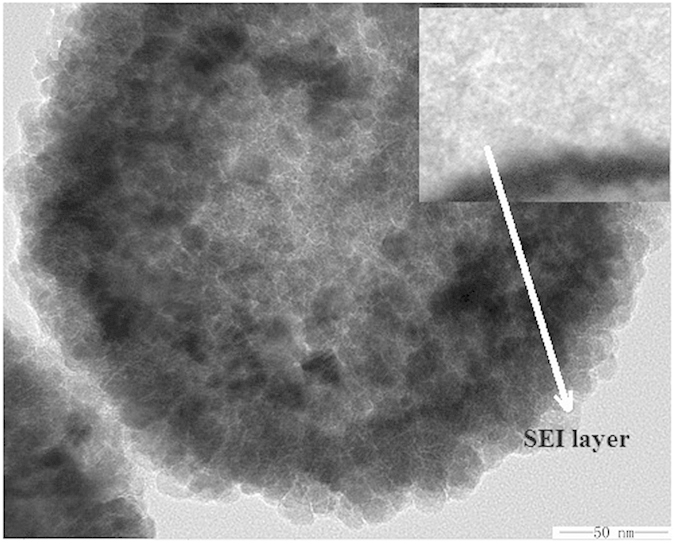
TEM image of hybrid hollow Fe_2_O_3_@MIL-101(Fe)/C electrodes after 200 cycles at current density of 500 mAg^−1^.

To investigate electrochemistry performance under the different rate discharge, Fig. 5d exhibits the discharge capacities of Fe_2_O_3_@MIL-101(Fe)/C electrode against different current density from 500 mAg^−1^ to 4000 mAg^−1^, and each sustained for 40 cycles. The stable cyclic performance is obtained for all densities. Even when the current density reaches 4000 mAg^−1^, the capacity can also arrive at 421 mAhg^−1^. Subsequently, a specific capacity of ca. 680 mAhg^−1^ is recovered when the current density reduces back to 500 mAg^−1^ after 200 cycles. The overall densities performance demonstrates the high capacities in both low and high current density of the hierarchical hollow Fe_2_O_3_@MIL-101(Fe)/C electrode. Figure 6 is the TEM of Fe_2_O_3_@MIL-101(Fe)/C electrode after 200 cycles at current density of 500 mAg^−1^, revealing materials is still kept well without breakage in the process of charge-discharge. SEI layer also can be detected accord to the inset in the Fig. 6, which is resulted from the decomposition of electrolyte. Compared with the previous reported iron oxide materials^1^,^7^,^11^,^15^,^32^,^33^, the material reported here is very attractive due to its facile, fast, and improved sodium storage. The nano-scaled characteristics of Fe_2_O_3_ particle from MOFs embedded in the aggregates ensure the electrode having a high capacity and the fast Na-ion diffusion in the electrode, and the introduction of carbon renders the electrode having a good electronic conductivity. The unique hollow structures can shorten the length of Na-ion diffusion, which is benefit for the rate performance. The hollow structure offers a sufficient void space, which sufficiently alleviates the mechanical stress caused by volume change. Therefore, the hierarchical hollow Fe_2_O_3_@MIL-101(Fe)/C electrode exhibits excellent electrochemical performance.

## Conclusions

Hierarchical hollow hybrid Fe_2_O_3_@MIL-101(Fe)/C materials have been successfully synthesized by a facile template-free procedure combined with a solvothermal synthesis reaction and subsequent calcination. As anode materials used in Na^+^ batteries, the stable reversible capacity of Fe_2_O_3_@MIL-101(Fe)/C electrode is 710 mAhg^−1^, and can be retained at 662 mAhg^−1^ after 200 cycles with the retention of 93.2%. This strategy is simple, cheap and suitable for mass-production, which may open a new avenue for fast synthesis of hollow or amorphous structural nano/micro-functional materials for energy storage, sensors, catalysts, and other new applications.

## Experimental section

### Materials and Methods

#### Synthesis of hollow Fe_2_O_3_ precursor

All chemicals were of analytical grade and used without further purification. Typically, FeCl_3_·6H_2_O (1.52 g), sodium citrate (3.02 g), urea (0.55 g) were dissolved in 80 mL deionized water and stirred for 30 min to form a homogeneous solution. Then 0.8 g PVP (MW30000) was added to the above solution with a continual stirring for 60 min. Subsequently, the light yellow solution was transfered into a 100 mL Teflon-lined stainless steel autoclave and held at 180 °C for 12 h. Finally, the black products were harvested through several rinse-centrifugation cycles with deionized water and absolute ethanol, and then dried at 60 °C under vacuum condition overnight.

#### Synthesis of Fe_2_O_3_@MIL-101(Fe)/C

To synthesis the Fe_2_O_3_@MIL-101(Fe)/C, 0.25 g the as-prepared hollow Fe_2_O_3_ precursor was dispersed in FeCl_3_·6H_2_O ethanol solution (4 mL, 10 mM) for 15 min, then in terephthalic acid ethanol solution (4 mL, 10 mM) for 30 min at 70 °C. The sample was collected by several rinse-precipitation cycles with absolute ethanol, and then dried at 120 °C under vacuum condition. The as-prepared Fe_2_O_3_@MIL-101(Fe) (0.45 g) was dispersed in 60 ml 0.5 M aqueous glucose solution, and then the solution was transferred into 100 mL Teflon cup. The autoclave was heated to 180 °C for 12 h. The resulting product was harvested by several rinse-precipitation cycles with methanol and was dried in vacuum at 60 °C for further characterization. Finally, certain amount of the MOFs precursors corresponding to the condition of hydrothermal treated at180 °C for 12 h were successively annealed in air flowing at 450 °C for 5 h with a slow ramp rate of 1 °C min^−1^ to make Fe_2_O_3_@MIL-101(Fe)/C microspheres.

### Characterization

X-ray diffraction (XRD) was carried out to identify the phase composition of synthesized samples over the 2θ range from 20° to 90° using a Rigaku D/max-A diffractometer with Co Kα radiation. A Fourier transform infrared spectroscope (FTIR, Themo Nicolet 670FT-IR) was used for recording the FTIR spectra of the sample ranging from 400 to 4000 cm^−1^. Morphologies of the synthesized samples were observed with a AMRAY 1000B scanning electron microscope (SEM), and the microstructural characteristics of samples were observed by high-resolution transmission electron microscope (HR-TEM, JEOL JEM-2010) working at 200 kV accelerating voltage and the lattice structure was identified by selected area electron diffraction (SAED) technique. Nitrogen adsorption-desorption measurements were conducted at 77 K on a Micromeritics Tristar apparatus. Specific surface areas were determined following the Brunauer-Emmet-Teller analysis.

### Electrochemical Measurements

For electrochemical performance evaluation, half-cell studies were performed. In the experimental electrode, acetylene black powder and polyvinylidene fluoride (PVDF) were used as conductive additive and binder. The working electrodes of sodium-ion batteries were fabricated by blending the active material, acetylene black and polyvinylidene fluoride (PVDF) in N-methyl-2-pyrrolidone with a weight ratio of 80:10:10. The obtained slurry was pasted onto a copper foil as current collector. After solvent evaporation, the electrode was pressed and dried at 120 °C under vacuum for 48 h. The cells were assembled in argon filled glove-box. Na metal and glass fiber were used as counter electrode and separator, respectively. The electrolyte was 1.0 M NaClO_4_ dissolved in ethylene carbonate/diethyl carbonate (EC/DEC, 1:1 by volume). Cycling tests were carried out at the charge and discharge current density of 100 mAg^−1^, in the voltage range of 0.05–3.0 V versus Na/Na^+^ by Land 2100A tester. Cyclic voltammetry was performed between 0.05 and 3.0 V with scan rate of 0.05 mVs^−1^.

## Additional Information

**How to cite this article**: Li, C. *et al.* Hierarchical hollow Fe_2_O_3_@MIL-101(Fe)/C derived from metal-organic frameworks for superior sodium storage. *Sci. Rep.* **6**, 25556; doi: 10.1038/srep25556 (2016).
